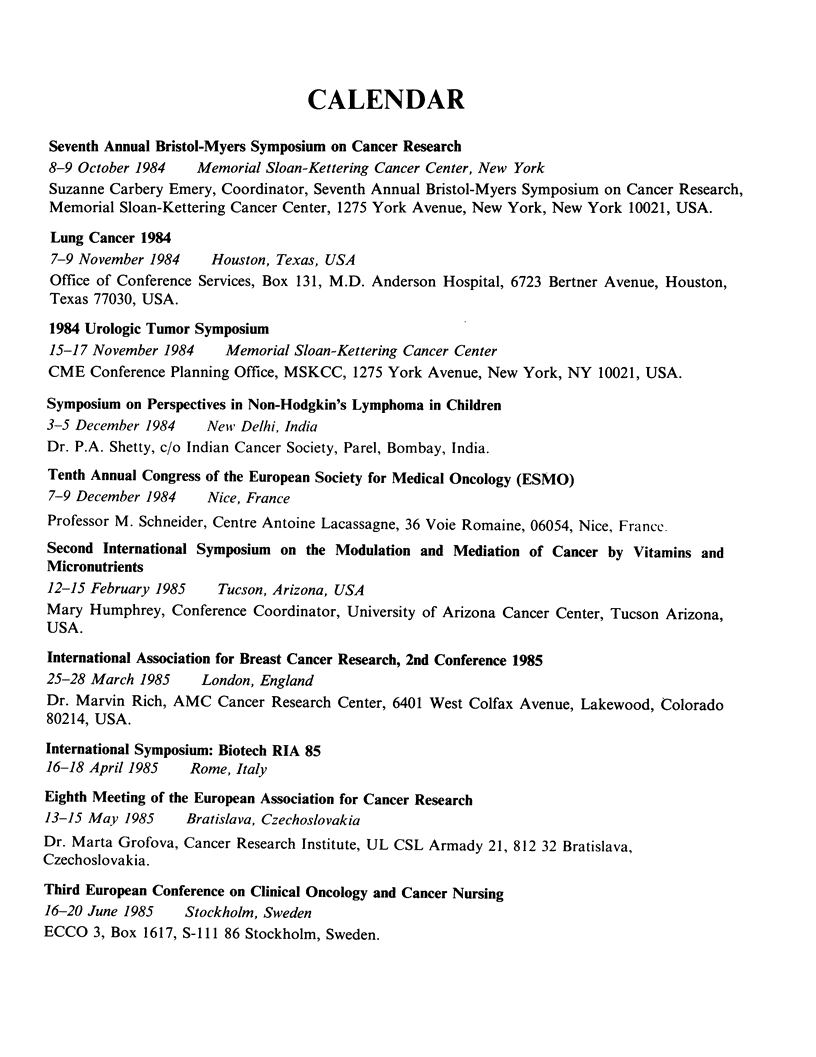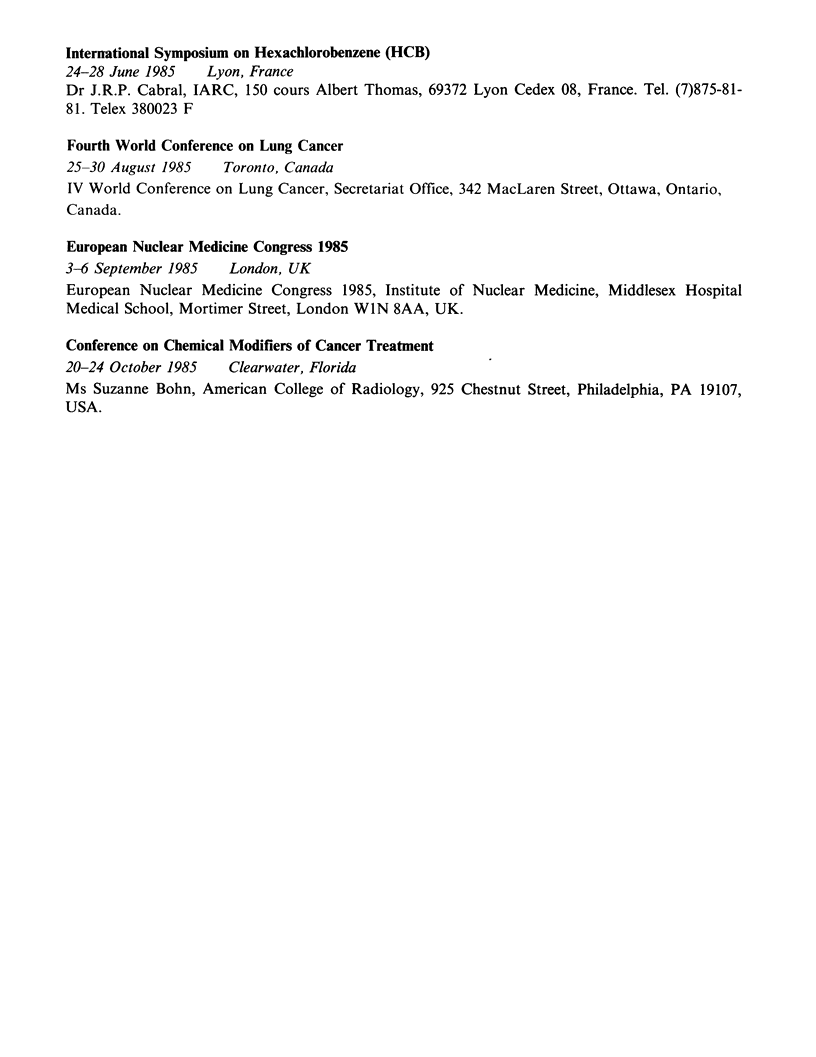# Calendar

**Published:** 1984-10

**Authors:** 


### CALENDAR

**Seventh Annual Bristol-Myers Symposium on Cancer Research**
*8–9 October 1984 Memorial Sloan-Kettering Cancer Center, New York*
Suzanne Carbery Emery, Coordinator, Seventh Annual Bristol-Myers Symposium on Cancer Research, Memorial Sloan-Kettering Cancer Center, 1275 York Avenue, New York, New York 10021, USA.

**Lung Cancer 1984**
*7–9 November 1984 Houston, Texas, USA*
Office of Conference Services, Box 131, M.D. Anderson Hospital, 6723 Bertner Avenue, Houston, Texas 77030, USA.

**1984 Urologic Tumor Symposium**
*15–17 November 1984 Memorial Sloan-Kettering Cancer Center*
CME Conference Planning Office, MSKCC, 1275 York Avenue, New York, NY 10021, USA.

**Symposium on Perspectives in Non-Hodgkin's Lymphoma in Children**
*3–5 December 1984 New Delhi, India*
Dr. P.A. Shetty, c/o Indian Cancer Society, Parel, Bombay, India.

**Tenth Annual Congress of the European Society for Medical Oncology (ESMO)**
*7–9 December 1984 Nice, France*
Professor M. Schneider, Centre Antoine Lacassagne, 36 Voie Romaine, 06054, Nice, France.

**Second International Symposium on the Modulation and Mediation of Cancer by Vitamins and Micronutrients**
*12–15 February 1985 Tucson, Arizona, USA*
Mary Humphrey, Conference Coordinator, University of Arizona Cancer Center, Tucson Arizona, USA.

**International Association for Breast Cancer Research, 2nd Conference 1985**
*25–28 March 1985 London, England*
Dr. Marvin Rich, AMC Cancer Research Center, 6401 West Colfax Avenue, Lakewood, Colorado 80214, USA.

**International Symposium: Biotech RIA 85**
*16–18 April 1985 Rome, Italy*

**Eighth Meeting of the European Association for Cancer Research**
*13–15 May 1985 Bratislava, Czechoslovakia*
Dr. Marta Grofova, Cancer Research Institute, UL CSL Armady 21, 812 32 Bratislava, Czechoslovakia.

**Third European Conference on Clinical Oncology and Cancer Nursing**
*16–20 June 1985 Stockholm, Sweden*
ECCO 3, Box 1617, S-111 86 Stockholm, Sweden.

**International Symposium on Hexachlorobenzene (HCB)**

*24–28 June 1985* *Lyon, France*

Dr J.R.P. Cabral, IARC, 150 cours Albert Thomas, 69372 Lyon Cedex 08, France. Tel. (7)875-81-81. Telex 380023 F

**Fourth World Conference on Lung Cancer**

*25–30 August 1985* *Toronto, Canada*

IV World Conference on Lung Cancer, Secretariat Office, 342 MacLaren Street, Ottawa, Ontario, Canada.

**European Nuclear Medicine Congress 1985**

*3–6 September 1985* *London, UK*

European Nuclear Medicine Congress 1985, Institute of Nuclear Medicine, Middlesex Hospital Medical School, Mortimer Street, London W1N 8AA, UK.

**Conference on Chemical Modifiers of Cancer Treatment**

*20–24 October 1985* *Clearwater, Florida*

Ms Suzanne Bohn, American College of Radiology, 925 Chestnut Street, Philadelphia, PA 19107, USA.